# Drug resistance in cancer

**DOI:** 10.1038/sj.bjc.6602821

**Published:** 2005-10-18

**Authors:** E Yagüe, S Raguz

**Affiliations:** 1 MRC Clinical Sciences Centre, Hammersmith Hospital Campus, Du Cane Road, London W12 0NN, UK

**Keywords:** apoptosis, cytochrome P450, breast cancer, leukaemia, ovarian cancer, P-glycoprotein

## Abstract

Cancer Research UK has recently sponsored a meeting, organized by the UK Medical Research Council, on cancer drug resistance. Several of the molecular mechanisms responsible for this clinical outcome, such as DNA interstrand crosslink repair, apoptosis evasion, cytochrome P450 and P-glycoprotein, were discussed. There was a special focus on leukaemia, breast and ovarian cancer, and the potential use of positron-emission tomography to study anticancer-drug resistance. The progress made in translating these findings to the clinic, like Gefitinib, P-glycoprotein phenotyping, or genome-wide analysis technology, was also discussed.

Approximately 50 scientists and clinicians working in the field of drug resistance gathered recently for a 1-day workshop at the Wolfson Conference Centre (Hammersmith Hospital, London) to discuss their latest results. The meeting was organised by the MRC Clinical Sciences Centre and was sponsored by Cancer Research UK. The workshop focused on both basic mechanisms and clinical applications, with a strong emphasis on translational research.

## BASIC MECHANISMS

It has been now widely demonstrated that normal primary cells become tumorigenic when expressing hTERT, SV40 proteins LT and ST, and oncogenic ras. We are using the step-wise model of tumorigenesis (Hahn and Weinberg, 2002) to determine whether the ability of human primary embryonic skin fibroblasts to develop drug resistance is exclusive to the transformed state or extends to premalignant cells. Interestingly, cells at early stages of the tumorigenesis process were able to develop doxorubicin-resistant derivatives. This demonstrates that, at least in this cell model system, the ability to acquire drug resistance is not a consequence of the accumulation of mutations that occur during the proliferation of a transformed cell, but it is an intrinsic characteristic that appears before the complete set of genetic transforming alterations. We are currently dissecting the minimal set of genetic alterations necessary to switch on the ability to acquire drug resistance, and dissecting the drug resistance signatures by microarray analysis.

Drugs that produce DNA interstrand crosslinks between the two complementary strands of the double helix, are among the most widely used and most effective anticancer agents. Victoria Spanswick (London) described a highly sensitive and robust method to determine DNA crosslinks in leukaemia, myeloma and solid tumour samples: the single-cell gel electrophoresis (Comet) assay. Cells isolated from melphalan-treated multiple myeloma patients showed between 42 and 100% repair of radiation-induced DNA crosslinks, whereas no repair was observed in cells from naive patients. These findings suggest that DNA interstrand crosslink repair may be an important mechanism of clinical resistance to melphalan in multiple myeloma (Spanswick *et al*, 2002). Spanswick and co-workers are currently determining whether molecules involved in the nucleotide excision repair and double-strand break repair pathways are implicated in this process.

The cytochrome P450 enzymes are a large family of constitutive and inducible haem-containing oxidative enzymes with roles in the metabolism of xenobiotics and steroid hormone synthesis. Georgia Pass (Dundee) described the use of a mouse model with a conditional deletion in the liver cytochrome P450 reductase (HRN) to study cyclophosphamide (CPA) toxicokinetics and therapeutic response. CPA is an anticancer prodrug that is dependent on cytochrome P450 for its therapeutic effectiveness. Pass and co-workers have demonstrated that in HRN mice the *in vitro* metabolism and intrinsic clearance of CPA was over six-fold lower than in wild-type animals. This, and other *in vivo* data (Pass *et al*, 2005), confirms that hepatic metabolism is the major route of CPA elimination and disposition. It is clear that this powerful model will undoubtedly produce many more advances in our understanding of drug metabolism. Morag McFadyen (Aberdeen) described some of the P450s that are specifically upregulated in cancer, their role in drug resistance and different therapeutic strategies. By far the most interesting is CYP1B1. This is the only P450 whose expression is not detected at all in normal human tissues. However, many kinds of cancer, like breast or ovarian, show activation of CYP1B1 expression. McFadyen described briefly the main strategies that are being used to tackle P450-associated drug resistance: (a) the use of prodrugs, (b) clinical inhibitors, and (c) immunotherapy; for a detailed review see McFadyen *et al* (2004). Since CYP1B1 is exclusively expressed in tumour cells, it is one of the most promising targets for the development of CYP1B1-specific prodrugs.

Another important mechanism of drug resistance is apoptosis evasion. Michael Seckl (London) is dissecting the mechanisms by which fibroblast growth factor 2 (FGF-2) protects small cell lung cancer (SCLC) cells from etoposide-induced cell death (Pardo *et al*, 2003). FGF-2 inhibits Smac but not mitochondrial cytochoromc *c* release upon exposure to etoposide. Smac/DIABLO promotes cytochrome *c*-induced activation of caspases by sequestering the inhibitor of apoptosis protein (IAP) family of caspase suppressors. In addition, FGF-2 signalling increases the translation of existing mRNAs such as *BCL2* and *BCLXL*, *XIAP* and cellular *IAP-1*. RNA interference of IAPs or 40S ribosomal protein S6K2BII prevented FGF-2- mediated rescue of etoposide killing in SCLC. Thus, FGF-2 protects SCLC cells from chemotherapeutic drugs via post-transcriptional regulation of several genes, providing multiple mechanisms for postmitochondrial survival via signalling through the SK6/MEK/mitogen-activated protein kinase pathway.

Some of the many mechanisms that the cells use to grow in the presence of a cytotoxic drug are exemplified by the work of Eric Lam (London) on the role of FoxO (*F*orkhead b*OX*-containing protein, *O* sub-family) family of transcription factors. Members of the FoxO family play a role in the downregulation of cellular responses normally elicited by growth factors activating the PI(3) kinase signal transduction pathway. In a panel of nine breast cancer cell lines, expression of FoxO1a and FoxO3a correlated with the expression of the proapoptotic FoxO target Bim, which was associated with paclitaxel-induced apoptosis. Gene reporter experiments in MCF-7 cells suggested that FoxO3a is responsible for the transcriptional upregulation of Bim. RNA interference specific for *FoxO3a* reduced the levels of Bim and inhibited apoptosis in paclitaxel-treated MCF-7 cells (Sunters *et al*, 2003). In leukaemia, FoxO3a regulates cyclin D2 expression. Inhibition of BCR-ABL by STI571 leads to FoxO3a activation, which in turn induces the expression of BCL6, culminating in the repression of cyclin D2 transcription through a STAT5 element in the cyclin D2 promoter (Essafi *et al*, 2005).

## LEUKAEMIA

In acute myeloid leukaemia (AML) there is a clinical correlation between P-glycoprotein (PGP) expression and chemotherapy treatment outcome and it is one of the most powerful prognostic factors. However, the clinical trials with competitive PGP antagonists have produced mixed and inconclusive results. According to Monica Pallis (Nottingham), a possible explanation for such mixed results may be due to the fact that no trials have been carried out with exclusively PGP-positive patients. Pallis and co-workers have developed sensitive and robust methods for the reproducible measurements of PGP in AML blasts permitting classification of the PGP status of AML patients in the clinical practice. Pallis' protocol involves a CD45 gate for blasts and the modulation of rhodamine 123 efflux by PGP with PSC 833. This functional analysis has proven more reliable than MRK-16 (a PGP-specific antibody) binding and has shown no intercentre variability (Pallis *et al*, 2005). Therefore, Pallis' methodology could now be translated to hospital immunophenotyping laboratories. From receiving a marrow sample, the assay takes about 4 h and can be carried out with as few as 3 million cells. Using this protocol, trial organisers would have the choice of whether to give PGP modulators to an unsorted cohort or to PGP-positive patients only.

Contrary to AML, PGP is not associated with drug resistance in childhood acute lymphoblastic leukaemia (ALL). Andy Hall (Newcastle) described the use of a single nucleotide polymorphism (SNP)-based chip to study loss of heterozygosity (LOH). Allelic imbalance is frequently observed in malignant cells and contributes to the deregulation of cell division and apoptosis through the deletion of tumour suppressor genes. Although other techniques such as karyotyping, comparative genomic hybridisation, or microsatellite analysis, have been used in the past, they are either of low resolution or laborious to conduct on a genome-wide scale. The recent introduction of oligonucleotide microarrays designed for the genome-wide typing of SNPs, with a resolution of 100–200 kb, has been used by Hall's group to characterize progressive LOH in samples from children with ALL who relapse after chemotherapy (Irving *et al*, 2005). Although Hall and co-workers have used a small number of patients in their preliminary study, their data suggest that progressive LOH may be a cause of disease progression and/or drug resistance. The most frequent abnormality detected was a loss of the *INK4* locus at relapse, suggesting that this abnormality may be commonly associated with treatment failure. Overall, Hall's results show that SNP array analysis is a powerful new tool for the analysis of allelic imbalance in leukaemic blasts.

## BREAST CANCER

Breast cancer accounts for one in four of all female cancers, making it by far the most common cancer in women in the western world. Around one in nine women in the USA and UK will develop breast cancer at some stage in their lives. Breast cancer treatment involves surgical removal of the tumour, but this is ineffective if malignant cells have escaped from the site of the primary tumour. Discovery of the involvement of the ovarian hormone oestrogen paved the way for the development of therapies that inhibit oestrogen synthesis or block its receptor. In many cases, however, these therapies fail due to recurrent endocrine-resistant tumours and much effort is being made to elucidate the mechanisms that underlie resistance to endocrine therapies. Iain Hutcheson's (Cardiff) group has developed an *in vitro* model system to dissect the altered signalling pathways in tamoxifen-resistant (TAMR) cells. Altered growth factor signalling, notably epidermal growth factor receptor (EGFR), c-erbB2, and insulin-like growth factor I receptor (IGF-IR) make a significant contribution to the development of antioestrogen resistance. As a consequence, several Phase II studies have been initiated examining Gefitinib (Iressa, a EGFR- selective tyrosine kinase inhibitor) monotherapy in TAMR breast cancer. Hutcheson has shown that TAMR cells that have become *in vitro* resistant to Gefitinib, are highly invasive, and that IGF-IR signalling is involved in these changes. Current efforts in Hutcheson's lab are aimed at using these cell model systems to test combination therapies to prevent the development of antioestrogen and antigrowth factor resistance (Nicholson *et al*, 2003).

Following the significant enhanced response of breast cancer patients to docetaxel neoadjuvant chemotherapy (Smith *et al*, 2002), Andrew Schofield's (Aberdeen) group is dissecting the mechanism that could mediate resistance to this drug in cell model systems by combining comparative genomic hybridisation (CGH) and expression profiling with cDNA microarrays. Several candidate genes: *PTEN*, *MDR1*, *Bax p27Kip1*, among many others, were highlighted (McDonald *et al*, 2005). The use of RNAi to downregulate *p27Kip1*, has indicated that docetaxel resistance in these cells is very likely the result of altered expression of multiple genes.

Genome-wide expression profiling is a powerful tool that is becoming increasingly important not only for the classification of tumours, but also for the prediction of clinical outcome. John Foekens (Rotterdam) described the use of gene expression profiling analysis in breast cancer. The promoter DNA methylation status of 117 genes was studied in a cohort of 200 steroid hormone receptor-positive tumours of patients who received the antioestrogen tamoxifen as first-line treatment for recurrent breast cancer. The methylation status of 10 genes was significantly associated with clinical outcome of tamoxifen therapy. Of these, *PSATI* was a predictor of tamoxifen therapy response (Martens *et al*, 2005). In another study, Foekens and co-workers have identified a specific signature for lymph-node-negative patients at high risk of distant recurrence. This is important because it would allow clinicians to avoid adjuvant systemic therapy or to choose less aggressive therapeutic options for lymph-node-negative patients who lack this signature (Wang *et al*, 2005).

## OVARIAN CANCER

Approximately, one out of 48 women in the western world develop epithelial ovarian cancer. In patients with organ-confined cancers, surgery alone is curative in more than 90% of cases. However, in most patients the tumour has disseminated beyond the ovaries by the time it is diagnosed; in these cases, combined treatment with surgery and chemotherapy is necessary, but acquired drug resistance is common (Agarwal and Kaye, 2003). Roshan Agarwal's (Sutton) research focuses on determining altered expression patterns in ovarian cancer drug resistance. Instead of using cell model systems, Agarwal is using patient biopsies analysed by a combined approach of CGH and expression prolifing with commercial BACs and expression microarrays. By using tumour cells from ascitic fluids purified by BerEP4, an epithelial marker antibody, Agarwal has obtained a 95% pure population of tumour cells suitable for expression profiling. Although the work is still in progress, preliminary results indicate that some of the candidate genes, like tubulin, had a previous association with paclitaxel drug resistance, thus validating Agarwal's methodology.

## PRECLINICAL APPLICATIONS

Catharine West (Manchester) discussed the potential of positron-emission tomography (PET) to study anticancer-drug resistance. At present, most of drug resistance-PET is limited to animal or preclinical studies. The most widely used PET imaging probe is the glucose analogue ^18^F-FDG whose uptake is used in the diagnosis to distinguish between benign and malignant lesions, and for the staging of cancer. 5-Fluorouracil (5-FU) is widely used as an anticancer treatment; however, drug resistance remains a significant problem that limits its efficacy. Pathways leading to 5-FU resistance include decreased uptake due to extracellular catabolism or poor tumour perfusion, increased efflux once inside the cell, increased intracellular degradation and metabolic inactivation. How and to what extent these different pathways contribute to drug resistance in patients and their variability is not known. 5-Fluorouracil labelled with ^18^F has been used in PET studies to increase the understanding of these mechanisms, which could be exploited in the further development of this drug or its analogues, or to aid the development of biomodulation approaches for increasing their efficacy (West *et al*, 2004).

## CONCLUDING REMARKS

The use of cell model systems, in spite of all their shortcomings, continue to be one of the main tools researchers have to identify candidate molecules and dissect the signalling pathways involved in drug resistance. In many cases, these basic studies lead to clinical trials such as Gefitinib for tamoxifen-resistant breast cancer.

Reproducible methodology has been developed to determine the PGP status of AML patients permitting the routine phenotyping of these patients.

Genome-wide analysis methodologies (CGH, SNP-arrays, and expression microarrays) are being used at increasing pace. In many cases, some of which were reported in this workshop, we have progressed from a purely descriptive status to the prediction of the outcome of therapy or of the development of recurrent disease by specific gene signatures. It is difficult to foresee how quickly these technologies will translate to the clinical practice but one of the major drawbacks, discussed informally at coffee time, is obviously their current cost.

Drug resistance is a complex and dynamic phenotype. Unravelling the basic mechanisms giving rise to this multifactorial phenomenon and translating these finding in the design of novel therapeutic strategies in the clinic is the next challenge that both scientists and clinicians working in this field have for the years to come.
